# Feelings-of-Warmth Increase More Abruptly for Verbal Riddles Solved With in Contrast to Without Aha! Experience

**DOI:** 10.3389/fpsyg.2018.01404

**Published:** 2018-08-13

**Authors:** Jasmin M. Kizilirmak, Violetta Serger, Judith Kehl, Michael Öllinger, Kristian Folta-Schoofs, Alan Richardson-Klavehn

**Affiliations:** ^1^Neurodidactics and Neuro Lab, Institute for Psychology, University of Hildesheim, Hildesheim, Germany; ^2^Memory and Consciousness Research Group, Clinic for Neurology, Otto-von-Guericke University, Magdeburg, Germany; ^3^Parmenides Center for the Study of Thinking, Pullach, Germany

**Keywords:** insight, problem solving, consciousness, feeling-of-warmth, intuition, cognition

## Abstract

When we are confronted with a new problem, we typically try to apply strategies that have worked in the past and which usually lead closer to the solution incrementally. However, sometimes, either during a problem-solving attempt that does not seem to lead closer to the solution, or when we have given up on problem-solving for the moment, the solution seems to appear out of nowhere. This is often called a moment of insight. Whereas the cognitive processes of getting closer to the solution are still unknown for insight problem-solving, there are two diverging theories on the subjective feeling of getting closer to the solution: (1) One that states that an intuitive feeling of closeness to the solution increases slowly, but incrementally, before it surpasses the threshold to consciousness and becomes verbalizable (=insight) (continuous approach), and (2) another that proposes that the feeling of closeness to the solution does not increase before it exceeds the threshold to consciousness (discontinuous approach). Here, we investigated the subjective feeling of closeness to the solution, assessed as feeling-of-warmth (FoW), its relationship to solving the problem versus being presented with it and whether a feeling of Aha! was experienced. Additionally, we tested whether Aha! experiences are more likely when the problem is solved actively by the participant or presented to the participant after an unsuccessful problem-solving attempt, and whether the frequency of Aha! experiences correlates with problem difficulty. To our knowledge, this is the first study combining the CRAT with FoW assessments for the named conditions (solved/unsolved, three difficulty levels, Aha!/no Aha!). We used a verbal problem-solving task, the Compound Remote Associates Task (CRAT). Our data revealed that Aha! experiences were more often reported for solutions generated by the participant compared to solutions presented after unsuccessful problem-solving. Moreover, FoW curves showed a steeper increase for the last two FoW ratings when problems were solved with Aha! in contrast to without Aha!. Based on this observation, we provide a preliminary explanation for the underlying cognitive process of solving CRA problems via insight.

## Introduction

Problems can be solved in many different ways, but one gross categorization of simple problems used in research is solving problems stepwise and analytically or by a sudden insight (Metcalfe and Wiebe, 1987). Analytical problem-solving refers to a gradual process of applying existing knowledge and available operators to a given problem representation. The best examples are probably mathematical equations for which one already knows the relevant formulas, or problems like the Tower of Hanoi. When prior knowledge fails to solve a problem, it is often necessary to turn away from known problem-solving approaches and invent something new. In such situations, people often get stuck in an impasse: a state of mind where the problem seems unsolvable. The driving force to overcome an impasse is thought to be a representational change, that either changes the given problem representation or the imposed goal representation (Ohlsson, 1992; Kershaw et al., 2013). A representational change is often accompanied by a deep insight into the solution of a novel problem. In our daily lives, such insights often occur when we have already turned our attention elsewhere, after being stuck with our unsuccessful problem-solving attempts for a very frustrating time. One of the earliest characterizations of insight proposes that a gap in the problem representation is detected and the problem solver is able to realize which components of the problem are essential for solving it (*selective encoding*), “synthesizing what might originally seem to be isolated pieces of information into a unified whole" (*selective combination*), and relating novel information to prior knowledge (*selective comparison*) (Davidson and Sternberg, 1984). Being able to realize which components of the problem are actually relevant for the solution is rather difficult for insight problems and is often thought to occur only after a representational change. Usually, those pieces of the problem are picked that seem the most promising based on prior experience (Knoblich et al., 2001). However, for insight problems, those are usually the ones that lead us into an impasse during our problem-solving attempt. A representational change needs to take place—the attentional focus needs to be shifted toward the actually relevant pieces of information which are usually less likely from our experience (Öllinger et al., 2014).

A recent study on representational change and insight assessed the dynamics of the representational change and whether they differ for problems solved with or without insight (Danek et al., 2018). The authors used videos of magic tricks and participants needed to figure out how they worked. Insight was operationalized as experiencing a subjective feeling of Aha! (solution being found suddenly, being confident it is correct). This operationalization has been frequently used since Jung-Beeman and colleagues introduced it (Jung-Beeman et al., 2004). The representational change was assessed by having participants rate the relevance of verbs for performing the tricks. The authors found that the shift from irrelevant to relevant verbs occurred gradually for no Aha! and more sudden for tricks solved with Aha!.

This pattern bears high similarity with the subjective feeling of closeness to the solution (Metcalfe and Wiebe, 1987; Reber et al., 2007; Hedne et al., 2016), sometimes operationalized as feeling-of-warmth (FoW, in the style of the children’s game pot hitting^1^). Metcalfe and Wiebe (1987) compared the dynamics of FoW during solving classical insight problems (problems which are thought to lead to an initial impasse during problem-solving), incremental problems (e.g., the Tower of Hanoi), and algebra problems. They found that FoW increased incrementally for non-insight problems and more suddenly for insight problems.

The likeness between the dynamics of the representational change and FoW for insight problems may suggest FoW as an intuitive marker of a representational change in the right direction. Intuition can be defined as the ability to comprehend an idea or being able to judge stimulus characteristics without being consciously aware of the knowledge on which this judgment is based (Ilg et al., 2007). Seeing FoW as an intuitive marker of the representational change would be in line with Bowers’ proposal that there are two stages of intuition: (1) a guiding stage, that is, the implicit perception of coherence of thought (intuition), and (2) an integrative stage during which the problem components form a plausible solution that is available to consciousness (insight) (Bowers et al., 1990, 1995). However, this approach on intuition and insight is in conflict with another approach that regards insight, intuition and analytical/incremental problem-solving as three different processes (Reber et al., 2007). Reber et al. (2007) propose that during analytical problem-solving, subjective and objective closeness to the solution increase equally linearly. In contrast, when a problem is solved by insight, the subjective feeling of closeness is at first level and only increases just before the solution becoming consciously available. How the objective closeness to the solution increases in the case of an insight solution, is not specified. The intuitive problem-solving process differs from the insight process by the objective closeness increasing linearly, while the subjective closeness raises at first linearly but with a flatter slope than for analytical problem-solving, and surges suddenly just before the solution becomes verbalizable. Reber’s model of intuitive problem-solving seems to map Bowers’ idea of intuitive problem-solving attempts that culminate in an insight (Bowers et al., 1990, 1995).

Zander et al. (2016) discussed the two approaches on insight in a review on insight and intuition. They described continuous and discontinuous models for both and conclude that intuition researchers favor the continuous model of intuition. In the continuous model, intuition is based on an early assessment of initial semantic search processes for the solution, culminating in an insight when the solution becomes accessible to conscious thought. In contrast, insight researchers seem to favor a discontinuous model which sees intuitive feelings about the correct solution as a misdirection of the problem-solving attempts that lead into an impasse, from which only restructuring may lead to an insight. Here, we consider FoW as an equivalent of an intuition about the closeness to the solution. On the first glance, the discontinuous model seems congruent with Reber’s model curve of insight. However, if intuition were to lead the problem solver astray, FoW should increase before the problem solver gets stuck in an impasse, only to decrease again, when the participant realizes that their intuition was incorrect. This process would probably be repeated several times before reaching a solution, resulting in a zigzag curve of FoW with a sudden final surge at the end^2^. If intuition were to culminate in insight, we would expect only one increase in the feeling of warmth, not an early increase followed by a decrease.

So far, we have only considered problems that are solved. What about problems that are not solved? Could insight also be involved when a solution is not found by the participant? There are very few studies we know of that looked at unsolved or incorrectly solved problems in the context of insight. Kizilirmak and colleagues report that Aha! experience are reported by participants also for unsolved problems for which the solution was presented (Kizilirmak et al., 2016a,c). However, a preceding attempt at problem solving seems important for the Aha! experience to occur, as it showed a higher prevalence for solutions that were presented after an unsuccessful attempt at problem solving (mean frequency == 0.41, *SD* = 0.14) as opposed to solutions that were immediately presented (0.31, *SD* = 0.35) (Kizilirmak et al., 2016c). Danek and Wiley (2017) investigated Aha! experiences for incorrect solutions and found that they were qualitatively different to Aha! experiences for correctly solved problems. That is, surprise was more strongly related to incorrectly solved problems with Aha!, whereas for correctly solved problems with Aha! it was tension relief. However, it is difficult to say whether the Aha! experience could be likened to insight or whether it is necessary for a problem solver to find the correct solution on his own, because there is no common definition of insight used by all insight researchers. Currently, however, most researchers think of solutions to problems that were solved with an Aha! experience as insight solutions, and this is what we will stick to in the present study.

### Aims of the Current Study

The current study investigates the dynamics of the subjective perception of closeness to the solution during verbal-problem solving separated by solutions solved either by the participant or presented after an unsuccessful attempt. This classification is detailed by reported Aha! problem difficulty. Until now, FoW dynamics were tested for classical single-trial insight problems (i.e., a set of very different problems) but without considering Aha! (Metcalfe and Wiebe, 1987) and with magic tricks for problems solved with versus without Aha! (Hedne et al., 2016). We would like to add to these findings by showing how the subjective perception of closeness to the solution develops over time for problems solved with Aha! and without Aha! and for solved versus presented solutions. So in line with this research topic’s aim of showcasing (a) either novel methods to research creativity or (b) the application of tried and tested methods in a novel way, the current study represents one of the latter.

We assessed FoW ratings and subjectively reported Aha! experiences while participants tried to solve Compound Remote Associate Task (CRAT) problems of three levels of difficulty. The CRAT is a verbal problem-solving task during which three words are presented that on first glance seem unrelated (e.g., power, shoe, radish). A fourth word needs to be found that can be used to form compound words with each of the other three (horse). The task is thought to be well suited to provoke insight solutions, because close associations with the three problem words often lead to an impasse (e.g., power outage, power rangers, power point,…).

The CRAT was originally developed by Bowden and Jung-Beeman (2003) who based their task on the Remote Associates Task by Mednick (1962) who intended this task as a test of students’ creativity. We believe that our study is a good extension of Hedne et al. (2016) in which magic tricks were used. We have shown that generating solutions to insight problems with Aha! are closely related to enhanced long-term memory for the problem and its solution (Kizilirmak et al., 2016a,c). The underlying mechanism is probably driven by reward-related processes. The sudden comprehension of difficult solutions is related to positive feelings such as tension relief (Danek et al., 2014), as well as the novel information (the solution) being easily integrated into prior knowledge (schema-based learning) (Kizilirmak et al., 2016b).

Gaining a better understanding of the dynamics of the subjective perception of closeness to the solution by means of FoW ratings will help us in understanding the cognitive process of insight, under which circumstances it occurs, and whether intuition can be seen as an antecedent of the Aha! experience, at least in the case of the CRAT. So far, this is the first study to use the CRAT for investigating FoW in general and in relation to the subjective feeling of Aha!.

Based on previous findings, we expected roughly equal distributions for generated and non-generated solutions. For FoW dynamics, we expected several potential outcomes: (a) Either a replication of Hedne’s and Metcalfe’s findings (Metcalfe and Wiebe, 1987; Hedne et al., 2016), that is, an almost level curve for problems solved with Aha! that rises very suddenly just before a solution is reported. Such a curve would also be in line with Reber and colleagues’ model curve of insight (Reber et al., 2007). (b) Or a slow rise followed by a much steeper slope just before the solution is reported. This would be in line with Reber’s intuition model which we consider as reflecting Bowers’ idea that insight is the second stage of intuitive problem-solving. Regarding item difficulty, we expected a higher frequency of Aha! for difficult items. This hypothesis was based on a study of insight reports from real life, which suggests that problems for which Aha! experiences were reported were mostly so difficult that problem solvers got stuck in an impasse for a long time and turned to other matters before suddenly realizing the solution (Klein and Jarosz, 2011).

## Materials and Methods

### Participants

Thirty-six healthy young adults (six male) participated in the study after providing written informed consent in accordance with the Declaration of Helsinki (World Medical Association, 2013). The study was approved by the Ethics Committee of the University of Hildesheim, Germany. Participation was voluntary and compensated via course credits. Median age was 20.5 years (range: 18–35 years). All had normal or sufficient uncorrected vision for reading the stimuli with ease, as tested by letting participants read the instructions aloud. Five participants were left-handed, the remaining 31 participants were right-handed. However, as all conditions were assessed within-subjects, and button-assignments were counterbalanced across participants, handedness should have no confounding effect.

### Stimulus Material

For each participant, we used 96 German CRAT items of a 144 item selection of our original 180 items used in earlier studies (Kizilirmak et al., 2016b,c). All CRAT items consist of four nouns, three words that make up the problem and one word that is the solution. The words are either nouns or color words. The solution word is one which can be used to form a compound word with each of the other three by appending it either as a prefix or suffix. To enable the investigation of the influence of item difficulty (i.e., the probability of an item to be successfully solved within the time limit), we categorized the items into three levels of difficulty: easy, medium, difficult. This categorization was based on data from a normative data sample (*N* = 20) collected at the Otto-von-Guericke University of Magdeburg, Germany. The 48 items with the lowest solution rate (primary sorting) and highest response time (secondary sorting, e.g. all items with a solution rate of 50 % were further ranked according to response time) were classified as “difficult,” the 48 items with the highest solution rate and lowest response time were classified as “easy,” and 48 items around the median solution rate were classified as “medium.” The remaining 36 items were not used in this study to ensure a more clear-cut difference between the difficulty levels.

The thus selected 144 items were divided into three sets (48 problems each) that were matched for probability to be solved (used to determine problem difficulty), to elicit a subjective Aha! response, and for plausibility according to a normative data sample that used a different set of 20 participants. For the current study, two sets were chosen, which item pools were chosen were counterbalanced across participants according to a reduced Latin square. From the 96 problems, six items (two of each of three levels of item difficulty) were drawn pseudo-randomly for six practice trials presented prior to the experiment proper. The third pool was not used. It should be noted that for each participant, plausibility, solution probability, and Aha! probability was equal, while specific stimulus characteristics like word frequency and emotional valence were counterbalanced across participants, thereby preventing any confounding effects of those factors.

### Design

We investigated alleged differences in the course of the subjective feeling of closeness to the solution (operationalized as FoW) depending on (1) whether the solution to a CRAT items was generated or presented after unsuccessful generation (factor = GENERATION), (2) whether the solution was comprehended with or without a feeling of Aha! (AHA), and (3) depending on item difficulty (DIFFICULTY). Participants were asked to assess their subjective closeness to the solution by means of a FoW on a 5-point heat scale (from 0 = white = cold to 4 = red = hot). FoW was assessed for the first time 6–7 s after stimulus onset to provide additional time for initial reading of the words, and every 4.5–5.5 s (pseudo-random jitter) thereafter until either coming up with a solution or reaching an upper time limit of 30 s (time for FoW ratings not counted). The jittered assessment time of FoW was intended to decrease the disturbance of the solution process by anticipated FoW ratings. The occurrence of an Aha! experience was assessed for each item after the solution was found or provided after reaching the upper time limit. Participants were required to decide via button press whether they had an Aha! experience or not.

### Task and Procedure

Firstly, participants were provided with oral and written information about the task and procedures as well as a consent form. After providing their written consent, they were asked to describe the task in their own words. This was done to check whether everything was understood as intended and to provide further instructions if necessary.

The main experimental task was conducted in a silent room with dimmed light inside a 1.3 deep, 4.0 m long, 2.0 m high box. The box serves as a shield against visual and partly auditory distractions. Participants were placed in a chair that was adjusted according to their height so that they could comfortably place their chin on a chin rest. The chin rest was placed exactly 1.0 m in front of a flat computer screen. The chin rest was part of a stationary 1250 Hz iView X eye-tracker (SensoMotoric Instruments, Teltow, Germany) with which we recorded additional gaze direction data which are, however, not part of the current report.

**FIGURE 1:**
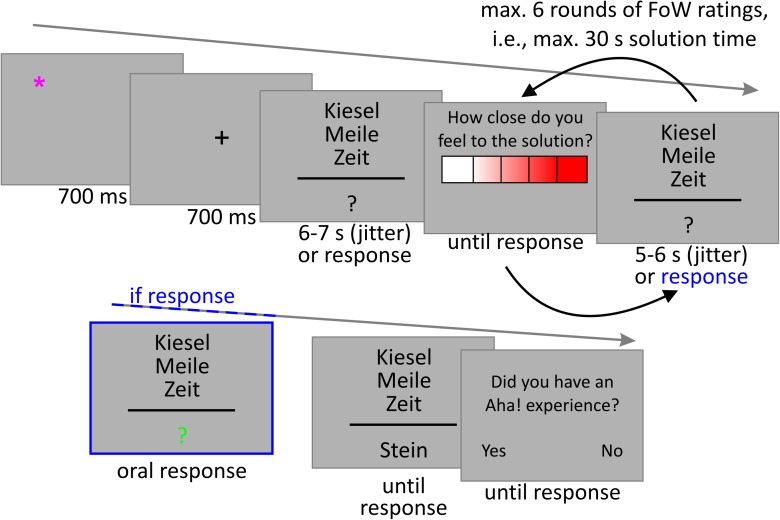
Exemplary trial. The problem words (top to bottom) can be translated to “pebble,” “mile,” “age,” and the solution word means “stone.” In case participants pressed the “solved” button during problem presentation, the question mark changed to green indicating that they should pronounce their solution. Otherwise, the solution was presented after the time limit.

Stimulus presentation and behavioral data collection was controlled via the software Presentation, version 20 (Neurobehavioral Systems, Inc., Berkeley, CA, United States). The task began with 6 practice trials, followed by a break and the chance to ask questions. The practice trials did not differ from the main trials. The 90 main trials were presented in three blocks a 30 trials. Before each block started, a 9-point (3 × 3 matrix, 800 × 800 pixels) calibration field for the eye-tracker was presented and participants were required to fixate on each point in turn as orally instructed while the experimenter calibrated the eye-tracker. During the breaks between blocks, participants were allowed to pace around. As depicted in **Figure 1** (exemplary trial), the background was always a medium grey (RGB code 178, 178, 178), the font Calibri, font size 28, font color black (RGB code 0,0, 0). During each trial, participants were presented with a star (^∗^) symbol that could appear in each of the four edges of an 100 × 100 pixels field centered on the screen. The position for the star was distributed equally and pseudo-randomly across trials. The star was presented in pink (RGB code: 255, 0, 127) for 700 ms. It was followed by a fixation cross presented in black (RGB code: 0, 0, 0) in the center of the screen for another 700 ms. Participants were instructed to first fixate the star and then shift their gaze to the cross as soon as it appeared. This procedure was implemented to support the synchronization of gaze direction data and behavioral data, because both were recorded by different computers. Directly after the fixation cross, the CRAT item without its solution was presented. The three triad words were stacked, centered, and 50 pixels apart in height. The third word was presented centrally. Below the three problem words, a question mark was presented as a place holder for the solution, separated from the problem words by a black line. Participants should press the space bar as soon as they came up with the solution for the problem. Each problem was presented for a total of 30 s or until participants pressed space to indicate that they came up with a solution. In case they did not press space, during the first 6 to 7 s (pseudorandom jitter), the first FoW rating had to be made. The question “How close to the solution do you feel?” was presented in German above a 5-point heat scale that consisted of five boxes (assigned range: 0 – 4), ranging from white (RGB code 255, 255, 255) to red (255, 0, 0) across different lighter tones of red. Participants could choose the corresponding via left and right arrow keys and should confirm via pressing the space bar. The next five FoW ratings were presented after 5–6 s (pseudorandom jitter), if the space bar was not pressed during the presentation of the problem. After reaching the upper time limit, the solution was presented in place of the question mark until participants pressed the space bar to indicate that they had understood how the solution word could be used to build compound words with all three triad words. In case participants indicated that they came up with the solution by pressing the space bar, the question mark changed color and became green (0, 255, 0), indicating that they should speak their solution out loud. The solution was then written down by the experimenter for data analysis. Either after providing a solution or after the solution was presented due to not solving the problem after 30 s problem presentation, participants were presented with the question “Did you have an Aha! experience? - Yes/No.” The left and right arrow keys were assigned Yes/No counterbalanced across participants. The Aha! experience was described in the written instructions in line with the four criteria proposed by Topolinski and Reber (2010): It was defined as the solution being comprehended suddenly, being convinced of the truth of the solution, feeling that the solution is easy to understand, once they know it. Moreover, it should be associated with a positive feeling. Like Bowden and Jung-Beeman (2003), we further emphasized that the described feeling of Aha! does not have to be overwhelming, but should closely correspond to this, because such laboratory insight tasks with a high number of trials of the same type will probably very rarely lead to the overwhelming feeling of Aha! in contrast to natural situations. At the end of the presentation, participants were asked to fill out a questionnaire that asked them about their strategies in solving the riddles and some other potential confounds, as well as demographic data. Median duration was 1 h 45 min (*SD* = 22 min).

### Data Analysis

Data were analyzed statistically using SPSS 24.0.0 for Mac OS (International Business Machines Corp., Armonk, NY, United States). We report conditioned probabilities in regard to the occurrence of Aha! given the solution was generated or not, once in regard to all items, and in regard to the number of FoW ratings per item. The number of rounds of ratings per item is dependent on how fast participants solved an item, as the FoW rating was given in intervals of 5–6 s, that is, 6–7 s for the very first round. All items with incorrectly generated solutions were excluded from data analysis, leaving only correctly generated and not generated solutions (relative number of excluded items: median = 0.08, *SD* = 0.07). In the following, when using the term “generated” we are always referring to correctly generated solutions. In case the distribution did not deviate from normality as tested via Kolmogorov–Smirnov test, non-parametrical tests were used, otherwise, parametrical tests were used. Effect sizes are reported as follows: Cohen’s *d* for repeated-measures *t*-tests and partial η^2^ for repeated-measures analyses of variance (ANOVAs). For Wilcoxon signed-rank tests, we $ES=\frac{z}{\sqrt{N}}$, as suggested by Pallant (2007), where *n* is the number of observations not participants. In case of a violation of the sphericity assumption as tested via Mauchly’s test, Greenhouse–Geisser corrected *p*-values and 𝜀 are reported together with uncorrected *F*-values and uncorrected degrees of freedom to enhance readability. In addition to effect sizes, we calculated the statistical power for each test *post hoc*. We did not use *a priori* power analyses for several reasons:

- We did not have any particular expectations about the effect size, as there is no prior feelings-of-warmth study using the Compund Remote Associates Task, and because having to reach, e.g., clinical relevance, no *a priori* threshold for power is necessary (besides, of course, for the effect reaching statistical relevance).
- We would have conducted the study even when we would have been unable to reach the optimal sample size, because strong effects would nevertheless be found, and those are the ones that are most likely reliable.
- Calculating the sample size for reaching a certain effect size typically does not take into account the fact that one may collect not one data sample per subject and per cell, but several, as we did. Estimating the true mean of the participant with several measurements per cell leads to a more accurate estimate and hence to a better estimate of the true population mean. Therefore, the power of such studies should also be higher. This is the standard procedure (to increase the number of trials per condition for each participant) for many psychophysiological, neuropsychological and Neuroscience studies, where it would take too many temporal, personal, and monetary resources to increase the sample size. However, as far as the authors are aware, standard power calculation tools like G^∗^Power provide no way to take this into account.

We therefore went along with a sample size that based on prior experience from numerous experiments led to large effect sizes. And indeed, as can be seen in our report of the statistical results, the minimum significant effect size was large.

Because it is highly discussed whether Aha! experiences can occur for non-generated solutions, that is, solutions that were presented after reaching the time limit without solving the problem, we also looked at the number of participants with empty cells for any condition.

## Results

### Frequencies of Conditions

Firstly, we computed the mean frequency of all combinations of GENERATION (generated, not generated), that is, whether a problem was solved or not solved), and AHA (aha, no aha), that is, whether participants reported an Aha! experience after they came up with a solution (generated) or after the solution was presented (not generated). All frequencies of conditions are listed in **Table 1**.

**Table 1:** Absolute (abs.) frequencies and conditional relative frequencies (rel.) of all conditions (without incorrectly generated items).

Condition	Min		Max		Mean		Standard deviation	
	abs.	rel.	abs.	rel.	abs.	rel.	abs.	rel.
P(aha | generated ∩ correct)	1	0.03	46	0.76	25.4	0.76	11.5	0.27
P(no aha | generated ∩ correct)	0	0.00	38	0.97	7.9	0.24	9.4	0.27
P(aha | non-generated)	0	0.00	59	1.00	27.1	0.57	14.5	0.29
P(no aha | non-generated)	0	0.00	52	1.00	21.0	0.43	15.1	0.29


**FIGURE 2:**
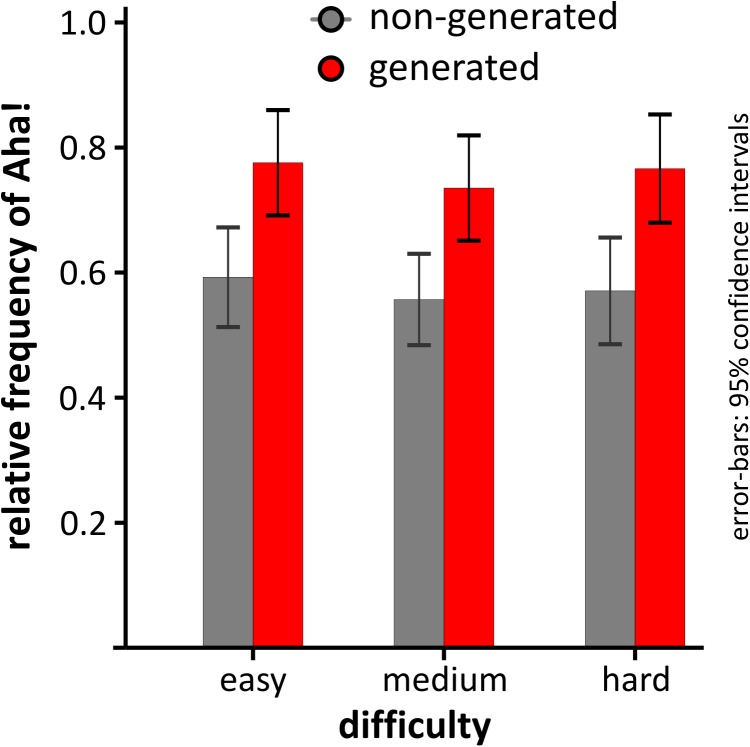
Relative frequency of Aha! depending on the level of difficulty and whether the solution to the problem was generated or not. Error-bars depict 95% confidence intervals corrected for repeated-measures (Masson and Loftus, 2003).

Another potential dependency we looked at was DIFFICULTY (easy, medium, hard). As can be seen in **Figure 2**, although the relative frequency of Aha! differed for generated and non-generated solutions, it did not differ according to problem difficulty. This observation was corroborated by a 2 × 3 repeated-measures ANOVA with the factors GENERATION and DIFFICULTY. As can be seen in **Figure 2**, there was a main effect of GENERATION [*F*(1,35) = 6.26, *p* = 0.017, $\eta_{p}^{2}$ = 0.152, power = 0.682], but no main effect of DIFFICULTY [*F*(2,70) = 1.71, *p* = 0.197, 𝜀_G-G_ =0.732, $\eta_{p}^{2}$ = 0.046, power_G-G_ = 0.295], nor an interaction [*F*(2,70) = 1.08, *p* = 0.875, 𝜀_G-G_ = 0.886, $\eta_{p}^{2}$ = 0.003, power_G-G_ = 0.065]. As reported in **Table 1**, significantly more Aha! experiences were reported for generated [*P*(aha| generated) = 0.76, *SD* = 0.27) compared to non-generated solutions [*P*(aha| non-generated) = 0.57, *SD* = 0.29), as tested via Wilcoxon signed-rank test [*T* = 179, *p* = 0.016, *ES* = 0.285, *power* = 0.654].

Secondly, we looked at the number of participants with empty cells, that is, zero cases of a certain combination of aha/no aha and generation/non-generation (see **Table 2**). There was only one participant who never reported Aha! experiences for non-generated solutions^3^. As can be taken from **Table 1**, Aha! experiences were reported for almost half of all problems that could not be solved. Interestingly, seven participants reported no case of solutions generated without Aha!, suggesting that the CRAT really might be more of an insight problem-solving task, that is, a task which is mostly solved via insight.

**Table 2:** Number of participants with zero cases per condition.

Condition	Number of participants
aha ∩ generated ∩ correct	0
no aha ∩ generated ∩ correct	7
aha ∩ non-generated	1
no aha ∩ non-generated	1


### Feeling-of-Warmth Course

The development of FoW can only be analyzed for items that were either not solved or solved after at least three rounds, because there is no curve otherwise. For items that were not solved, it will be interesting to see, whether participants felt closer to the solution by the end of the six rounds of FoW ratings or rather the 30 s of attempting to generating a solution.

#### Feeling-of-Warmth for Solved Items (Generated Solutions)

**FIGURE 3:**
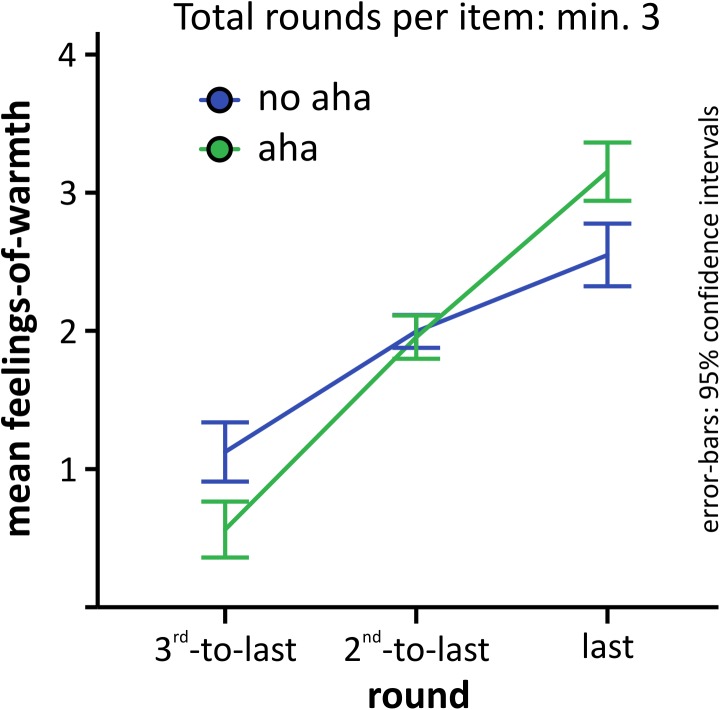
Development of the mean FoW for the last three rounds of all problems solved in at least three rounds. Error-bars as described for **Figure 2**.

First of all, we looked at the last three rounds of any item that was solved after at least three rounds and compared FoW curves for items solved with versus without Aha!. All participants could be included, because all of them had at least one trial solved within three rounds. The mean number of trials was 4.0 (*SD* = 4.1) for no aha and 10.6 (*SD* = 5.5) for aha. As can be seen in **Figure 3** and conform with the idea that FoW would increase suddenly when the problem is solved via insight (i.e., with Aha! experience), the curve for problems solved with Aha! was below the one solved without Aha! for the third to last round, but increased highly and above those solved without Aha! for the last round, just before the solution was found. We computed a 3 × 2 repeated-measures ANOVA with factors ROUND(third-to-last, second-to-last, last) and AHA(aha, no aha) to compare mean FoW ratings, and found a highly significant main effect for ROUND [*F*(2,52) = 132.27, *p* < 0.001, 𝜀 _G-G_ =0.845, $\eta_{p}^{2}$ =0.836, power_G-G_ =1.0], no main effect of AHA [*F*(1,26) = 1.05, *p* = 0.315, $\eta_{p}^{2}$ = 0.039, power = 0.167], and a highly significant interaction [*F*(2,52) = 15.63, *p* < 0.001, 𝜀_G-G_ =0.642, $\eta_{p}^{2}$ =0.375, power_G-G_ =0.988]. When comparing the difference between the means of the last minus third-to-last FoW ratings for problems solved with (2.59, *SD* = 0.97) versus without Aha! (1.43, *SD* = 1.07), we found a highly significant difference [*t*(26) = 4.27, *p* < 0.001, Cohen’s *d* = 0.821, *power* = 0.956], suggesting that the offset between the last and third-to-last FoW ratings may be a good marker for whether problem-solving is accompanied by a feeling of Aha! or not.

Secondly, we looked at FoW curves depending on the number of rounds needed until the solution was generated, and again compared them for items solved with versus without Aha!. We could only analyze problems solved within three (20 participants could be included, mean number of trials with aha = 4.3, *SD* = 2.3, mean number of trials with no aha = 2.15, *SD* = 1.7), four (13 participants, aha = 3.1, *SD* = 1.9, no aha = 1.9, *SD* = 1.2) and five rounds (9 participants, aha = 2.8, *SD* = 2.5, no aha = 1.8, SD = 1.4). This pattern, i.e., that most participants solved most items within the first three rounds, is typical for the CRAT, as Bowden and Jung-Beeman (2003) report that CRAT items are mostly solved within the first 15 s, which corresponds to three rounds in our design. Due to the low number of participants, we refrained from statistical inference testing, but report the data descriptively.

**FIGURE 4:**
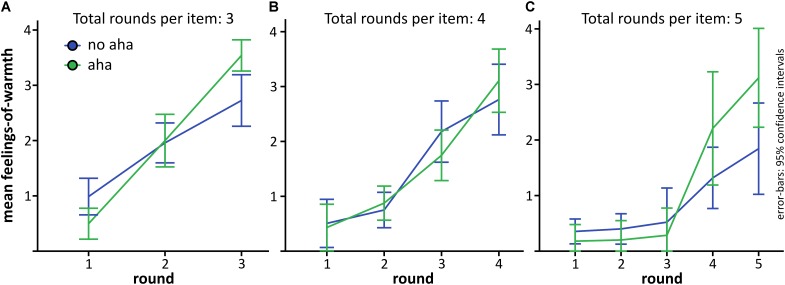
Development of FoW depending on the number of rounds per problem until the solution was generated. **(A)** Problems solved after 3 rounds. **(B)** Problems solved after 4 rounds. **(C)** Problems solved after 5 rounds. Problems solved after 6 rounds are not depicted, because the number of participants who had at least one problem solved during the last round was very low. Error-bars as described for **Figure 2**.

The pattern for problems solved within three rounds (**Figure 4A**) was highly similar to the pattern reported above and is in line with the idea that FoW rises suddenly for problems solved with aha. The curve for five rounds (**Figure 4C**) is also in line with this hypothesis, whereas the curves for items solved within four rounds (**Figure 4B**) seem to completely overlap for aha and no aha. The curves for four and five rounds suggest that the slope of the FoW curve is more of a second order polynomial function (tested with the curve fitting tool from https://mycurvefit.com, access date: 2018-03-28) rather than linear (as might be inferred from the three-point curves), in line with the model suggested by Reber et al. (2007).

#### Feeling-of-Warmth for Unsolved Problems (Non-generated Solutions)

**FIGURE 5:**
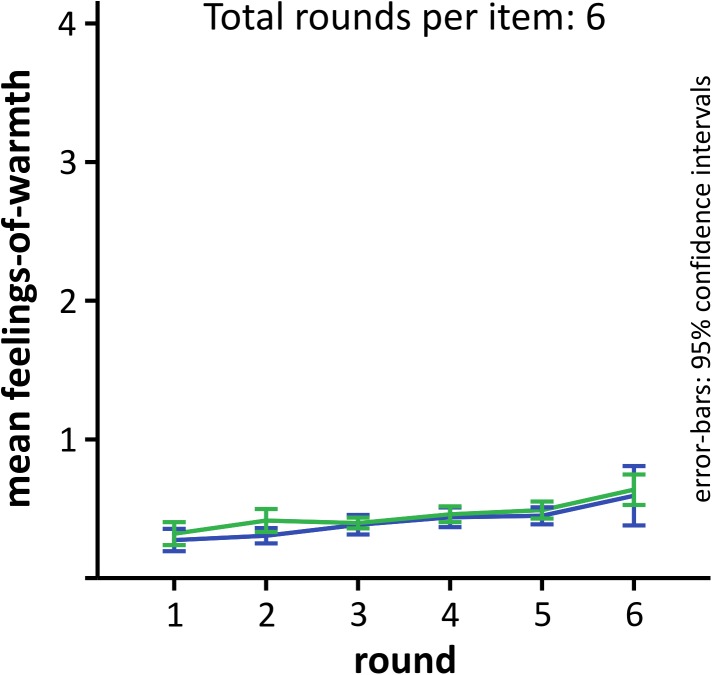
Development of FoW for unsolved problems for comparison. The Aha!/no Aha! decision was based on the solution that was presented after time-out. Error-bars as described for **Figure 2**.

For comparison, we also analyzed the development of FoW over time for unsolved problems, and compared the curves for problems solved with versus without Aha!. Thirty-four participants could be included in this analysis. Two participants had empty cells (one only reported Aha! experiences for non-generated solutions and the other only no Aha!). As expected, the curves show a flat course and did not differ for aha and no aha (**Figure 5**). A 6 × 2 repeated-measures ANOVA revealed a significant main effect of ROUND [*F*(5,165) = 9.78, *p* < 0.001, 𝜀_G-G_ = 0.393, $\eta_{p}^{2}$ = 0.229, power_G-G_ = 0.977], but no main effect of AHA [F(1,33) = 1.66, p = 0.207, $\eta_{p}^{2}$ = 0.229, power = 0.239], nor was there a significant interaction [F(5,165) = 0.342, p = 0.666, 𝜀_G-G_ = 0.323,$\eta_{p}^{2}$ = 0.010, power_G-G_ = 0.097]. There was a low but significant increase of FoW over time, although it stayed between the lowest two values (0, 1), suggesting that participants did never feel particularly close to the solution, before it was presented.

## Discussion

The present study investigated the relationship between the subjective closeness to the solution, assessed as FoW ratings, the subjective Aha! experience, item difficulty, and the generation of solutions for CRAT problems. This is the first study to investigate the relationship between a measure of the subjective closeness to the solution (FoW) depending on whether an insight occurred or not (feeling of Aha!).

### Feeling-of-Warmth Differ for Problems Solved With Versus Without Aha!

The observed FoW curves for problems solved in at least three rounds of 5–6 s each showed that insights, operationalized as experiencing a feeling of Aha! upon solving a problem, were characterized by a curve that showed a sudden increase of FoW during the last two FoW ratings (<10 s) before reporting a solution. The slope was much steeper for problems solved with than without Aha!. This finding is in line with an observation made by Metcalfe and Wiebe (1987) who measured FoW for solved insight problems as compared to analytical problems. However, as the authors defined insight problem-based and not process-based, we have to be careful when comparing their results with our findings. In terms of the continuous and discontinuous approaches on insight described by Zander et al. (2016), our results seem to be more in support with the continuous model, which proposes a slow increase that ends in a sudden surge, similar to the curve proposed by Reber et al. (2007) for intuitive problem solving and we conceive a curve that depicts Bowers’ approach on insight as the final stage of intuitive problem-solving (Bowers et al., 1990). However, because we have only enough trials with at least three FoW ratings and because FoW was assessed in intervals of 5–6 s, our curve is not fine-grained enough to say for sure whether the FoW development is more similar to Reber’s intuition curve or his insight curve for the subjective closeness to the solution. Those two model curves only differ in regard to whether the slope is level (insight) or whether it rises just a little (intuition) before culminating in a sudden surge just before the solution is found. What we can derive with certainty from our data is that problems solved with Aha! do show more of a sudden increase at the end and those solved without Aha! show more of a gradual rise. Especially the curve with five FoW ratings suggests that there is a very sudden increase in FoW for problems solved with as compared to without Aha!. Although we have only few participants that solved problems after five FoW assessments, this suggests that if we were to assess FoW in a more continuous way, it would be in line with the insight model curve by Reber et al. (2007).

We propose that the observed FoW curves support the following cognitive process for insight solutions: When searching for the remote association that comprises the solution word of a CRA problem, the remote associations activated by means of spreading activation are at first not available to consciousness (see Öllinger and von Müller, 2017, for an alleged model of the underlying search process—combing spreading activation and constraint satisfaction). However, at the time when the associations are set up between all triad words and the solution word, its activation level becomes strong enough to become consciously available. This comprises the moment of Aha!.

Our findings are in contrast to those of Hedne et al. (2016) who measured FoW for magic tricks solved either with Aha! or without. They found no difference in FoW ratings (differential measure = last – first rating, angular measure = differential warmth/s) for tricks solved with or without Aha!. An important difference between Hedne and colleagues’ and our study is the frequency of Aha! for solved problems. Whereas for our task 76% of all solved items were solved with Aha!, Hedne and colleagues report almost the reverse distribution, namely 29% of all solved items were solved with Aha!. The low number of problems solved via insight may have led to a less accurate estimation of the true mean of FoW, not allowing to find differences between FoW for insight and non-insight solutions, even if there were any. This low frequency of Aha! for magic tricks seems a little surprising at first, because Danek and colleagues, who pioneered magic tricks as a task to investigate insight problem solving, always report higher distributions: 41.1% (Danek et al., 2013b), and 66.5% (Danek and Wiley, 2017). However, Hedne et al. (2016) reported not the Aha! rate for all correctly solved items, as Danek et al. (2013b) and we did, but Aha! for all solved items (be it correct or incorrect) (personal communication with Hedne, 2018 March 25). So, to make our reported Aha! rate more comparable across studies, we additionally calculated *P*(Aha! | generated(correct ∩ incorrect)) which was 72.9 % (SD = 21.6) and still deviated considerably from the other studies. There are other potential explanations of the diverging findings, such as differences of the Aha! definition participants were provided with, or that the tasks really differed considerably in their probability to induce an Aha! experience. Hedne et al. (2016) indeed defined the Aha! experience by only one criterion, that is, that the solution appeared “out of nowhere,” whereas the current study and Danek and colleagues included at least two of the four criteria suggested by Topolinski and Reber (2010): suddenness, being convinced of the truth of the solution, ease of understanding, and positive affect.

All in all, our findings support the idea that subjective feelings of closeness to the solution rise more suddenly for insight than for no insight. Moreover, they show the importance of how insight is defined (experimenter-based, participant-based) and if the participant-based approach is chosen, how the Aha! experience is described to the participants, when investigating differences in FoW curves for insight and no insight solutions. In terms of a more fine-grained differentiation between intuition, insight, and incremental problem solving as proposed by Reber et al. (2007), we unfortunately cannot draw any clear conclusions, because we ended up with too few trials for a statistical comparison between detailed FoW curves (4–5 ratings). It may be advised for future studies on the topic, to increase the number of trials.

### The Aha! Experience Is Related to the Generation of a Solution but Not Problem Difficulty

We found that Aha! experiences were more often reported when CRAT problems were solved compared to when the solution was comprehended only after failing at generating it (76% versus 57%). However, Aha! experiences were still reported relatively often even for presented solutions, suggesting that insight-like experiences can even be felt when comprehension is induced. Another study using CRAT problems reported Aha! frequencies of 56% for correctly solved items (Jung-Beeman et al., 2004). Unfortunately, there is no published data from other labs on Aha! rates for solutions to problems that were presented after a failed solution attempt. Importantly, we are not referring to problems that were solved incorrectly, but problems for which no solution was generated within the time limit. In previous studies, we observed an equal distribution of Aha! for generated and non-generated solutions for Mooney stimuli, that is, pictorial riddles (Kizilirmak et al., 2016a), or the reverse pattern, that is, a higher frequency of Aha! for non-generated CRAT problems (Kizilirmak et al., 2016c). However, either the stimulus material differed considerably (verbal semantic problems here versus pictorial visual problems in Kizilirmak et al., 2016a) or the conditions used (solution process repeatedly interrupted at short intervals and only problems where participants had the chance to solve them here versus problems with or without the chance to solve them in Kizilirmak et al., 2016c). It is therefore difficult to compare our results. The diverging findings for Aha! rates of correctly solved CRAT problems nonetheless suggest that there are many different factors aside from the problem type that play a role in whether items are solved with or without Aha!.

In contrast to our hypothesis, the frequency of Aha! experiences was not dependent on the difficulty level of the CRAT problem. In other words, whether the solution to a difficult, medium, or an easy CRAT problem is comprehended, the probability of experiencing an Aha! moment was equal. This observation complements the observations made by Knoblich et al. (1999) who found a relationship between task difficulty and the probability of a representational change. In matchstick arithmetic tasks the degree that a chunk decomposition or a constraint relaxation requires determined the solution rates and solution times. Given this evidence, our results suggest that problem difficulty of the CRAT is not exclusively caused by the degree of representational change but by an additional source of problem difficulty such as semantic distance, that is not related to the feeling of aha!. This interpretation is in line with the multiple causes of difficulty approach (Kershaw and Ohlsson, 2004; Kershaw et al., 2013; Öllinger et al., 2014).

On the other hand, it could also be that the variation of problem difficulty for CRAT problems was too low to enable us to find any significant differences between difficulty levels and Aha! frequency even if they existed. Other studies which quantified the Aha! rather than recording binary occurrence, report significant correlations between the strength of the Aha! experience and solution rates (as an operationalization of problem difficulty). For example, Webb and colleagues report significant but weak correlations [*r*(99) = 0.26–0.27) between solution rates (accuracy) and Aha! ratings of classic insight problems (such as the rope problem) and also for an English version of the CRAT (Webb et al., 2017). Danek and colleagues further observed significant differences for mean Aha! ratings of correct versus incorrect solutions (Danek et al., 2013b; Danek and Wiley, 2017). Hence, it may be that only the strength of the Aha! is related to problem difficulty, similar to the complexity of the representational change required (Knoblich et al., 1999), but not whether it occurs or not. Future studies should focus on tasks with a larger variability between task difficulty and assess solution rates as well as Aha! rates and the strength of the Aha! to test this assumption.

### Limitations

There are several limitations for the conclusions that can be drawn from the current manuscript. First, we do not know in how far our results can be generalized to other types of problems besides the CRAT and probably the incoherent triads that Zander et al. (2016) referred to in their review. Second, to assess the course of FoW, we interrupted the problem-solving process of our participants in intervals of 5–7 s. We do not know in which way this or even asking for a FoW rating in itself may influence the ratings. What we noticed is that the frequency of reported Aha! experiences differs from our other experiments using the CRAT with the same time for solving the problems (30 s in total). As we reported in 2016 in the Journal of Problem Solving, 24% of all items were solved with Aha!, 21% solved without Aha!, 41% were not solved with Aha!, and 14% were not solved without Aha! (Kizilirmak et al., 2016c). Thus, it looks like there may be an influence of the interruptions or the FoW ratings *per se*. However, as the paradigm also differed in the conditions present, because in the 2016 study, we had items for which participants had the chance to solve CRA items and those whose solutions were presented immediately, we cannot be sure that the diverging findings are only due to the interruptions or consciously considering the subjective closeness to the solution, as they might also be due to not having a no-chance to solve condition.

## Conclusion

Our results provide support for the idea that insight solutions pop into awareness suddenly, probably around 5–12 s before being able to indicate behaviorally that the problem has been solved. The slope for the last three FoW ratings (5–6 s apart) was significantly steeper for problems solved with Aha! compared to those without, lending support to the idea that the subjective feeling of closeness to the solution does not rise or only rises weakly until the solution is verbalizable. It is even conceivable that participants would be able to voice the solution at the time of the second-to-last FoW rating which is much higher than the third-to-last for insight, but only press the button after they have confirmed that their solution is a valid compound word for the three words comprising the CRA item. Future studies could instruct participants to voice a solution whenever they have a candidate, even when they are unsure, in addition to assessing FoW ratings, to test this hypothesis. We further found that CRA problems are mainly solved via insight (i.e., accompanied by a subjective feeling of Aha!) and that insight solutions do not depend on problem difficulty. This finding is very useful in regard to learning from insight, as other studies have shown that solving problems by insight facilitates long-term memory encoding (Danek et al., 2013a; Kizilirmak et al., 2016a): It is not necessary for the problem to be especially difficult to be solved with an Aha! experience. Hence, for the application of learning from insight, even easy problems can be used.

## Ethics Statement

The study was approved by the Ethics Committee of the University of Hildesheim, Germany. Participation was voluntary and compensated via course credits. All participants gave their informed written consent and were explicitly told that they could abort the procedure at any time without the need for an explanation and without any negative consequences.

## Author Contributions

JMK, A-RK, and KF-S conceived and designed the study. JMK programmed the experiment and additional scripts for later data analysis. JK carried out a pilot study under JMK’s and AR-K’s supervision (Bachelor’s thesis in Psychology) in A-K’s lab. VS conducted the main study under JMK’s supervision (Master’s thesis as a pre-service teacher) in KF-S’s lab. JMK, JK, and VS carried out the statistical analyses. JMK wrote the first draft of the manuscript. JMK, MÖ, A-RK, and KF-S revised the manuscript. All authors read and approved the submitted version.

## Conflict of Interest Statement

The authors declare that the research was conducted in the absence of any commercial or financial relationships that could be construed as a potential conflict of interest.

## Footnotes

1 This game is traditionally played by hiding a prize underneath a pot. Of a group, one is designated the seeker, blindfolded, and equipped with a wooden spoon. The seeker needs to try and find the pot by hitting the floor (and eventually the pot) while the others call “cold,” “warmer,” “hot,” depending on how close the seeker is to the pot. At least in Germany it is very famous (German name “Topfschlagen”).

2 Unfortunately, to accurately assess and map such a development, we would probably need more continuous FoW assessments, which is why we did not include this model as part of our testable hypotheses.

3 This number further seems to depend on how long it took participants to solve an item, because for items solved within three rounds, there were 14 participants who never reported no Aha!, for four rounds, there were 18, and for 5 rounds there were 23, while the number of participants who never reported Aha! experiences for solved items with 3, 4, and 5 rounds was very low and always the same 2. However, closer evaluation of their post-experimental questionnaires revealed no striking differences to the other participants.
